# Laryngeal microsurgery under Transnasal Humidified Rapid Insufflation Ventilatory Exchange

**DOI:** 10.1002/oto2.125

**Published:** 2024-06-11

**Authors:** Tiffany Rigal, Robin Baudouin, Marta Circiu, Florent Couineau, Jérôme Lechien, Lise Crevier‐Buchman, Morgan Le Guen, Stéphane Hans

**Affiliations:** ^1^ Department of Otolaryngology–Head and Neck Surgery Foch Hospital Suresnes France; ^2^ School of Medicine, UFR Simone Veil Université Versailles Saint‐Quentin‐en‐Yvelines (Paris Saclay University) Montigny‐le‐Bretonneux France; ^3^ Department of Otolaryngology Elsan Polyclinic of Poitiers Poitiers France; ^4^ Department of Human Anatomy and Experimental Oncology, Faculty of Medicine, UMONS Research, Institute for Health Sciences and Technology University of Mons (UMons) Mons Belgium; ^5^ Division of Laryngology and Broncho‐Esophagology EpiCURA Hospital Baudour Belgium; ^6^ Phonetics and Phonology Laboratory (UMR 7018 CNRS, Université Sorbonne Nouvelle/Paris 3) Paris France; ^7^ Department of Anesthesiology, Foch Hospital School of Medicine Suresnes France; ^8^ Simulation Center Foch Hospital Suresnes France

**Keywords:** airways management, apneic oxygenation, carbon dioxide laser, laryngeal microsurgery, laryngoscopy, THRIVE

## Abstract

**Objective:**

Since 2015, Transnasal Humidified Rapid Insufflation Ventilatory Exchange (THRIVE) has been used in general anesthesia for preoxygenation or difficult exposure airway management. Its use offers new opportunities in laryngology. THRIVE increases apnea time and frees the access to the upper airway. However, its use may be less stable than orotracheal intubation. The main objective of this work was to evaluate the feasibility of laryngeal microsurgery under THRIVE including using Laser.

**Study Design:**

Retrospective.

**Setting:**

A total of N = 99 patients with laryngeal microsurgery (with or without CO_2_ laser) under THRIVE were included successively from January 1, 2020 to January 30, 2022.

**Method:**

Medical history, comorbidities, clinical and surgical data were extracted and analyzed. Two groups were constituted regarding the “success” (use of THRIVE along all the procedure) or the “failure” (need for an endotracheal tube) of the use of THRIVE during the procedure.

**Results:**

A failure occurred in N = 15/99 patients (15.2%) mainly due to refractory hypoxia. The odd ratios (OR) for THRIVE failure were: OR = 6.6 [2.9‐35] for overweight (BMI >25 kg/m^2^); OR = 3.8 [1.7‐18.7] for ASA score >2; OR = 4.7 [2.3‐24.7] for the use of CO_2_ laser. Elderly patients and patients with pulmonary pathology were not statistically at greater risk of THRIVE failure. No adverse event was described.

**Conclusion:**

This work confirms the feasibility of laryngeal microsurgery under THRIVE, including with CO_2_ laser. Overweight, ASA >2 and lower fraction of inspired oxygen during CO_2_ laser use increased the risk for orotracheal intubation.

Laryngeal microsurgery requires excellent cooperation between anesthesia and surgical teams. New availability of short‐acting anesthetic agents in the 1990s and of the curare reversal agent sugammadex in the 2000s allowed the association of deeper anesthesia for shorter procedures with rapid recovery, more suitable for laryngeal microsurgery.^1^ Mechanical ventilation using an endotracheal tube as an interface is the gold standard of ventilatory control during procedure.^2^, ^3^ The main disadvantages of the endotracheal tube are that it causes a visual amputation of the operating field by masking the posterior part of the larynx, annoying the surgeon during laryngeal microsurgery particularly for posterior glottic lesions. The vision of one‐half of the glottic plane or the vision of the posterior part of the larynx may be compromised with detrimental consequences for the accuracy of the surgical procedure and risk for disease control (for inadequate resection).

Alternative modes to provide oxygenation have been developed, each with advantages and disadvantages. No single modality offers a complete solution to the problem. Apneic anesthesia with intermittent ventilation has been shown to be safe for the patient.^4^ Moreover, with this technique, the surgical field is freed. However, apneic anesthesia with intermittent ventilation is only suitable for the shortest procedures or the procedures must be paused to ventilate the patient, increasing at the end the operating time.

High‐frequency jet ventilation has been used since the 1970s to deliver oxygen or most commonly O_2_‐enriched air, with a low tidal volume with high velocity through a small diameter injector, far much thinner than an endotracheal tube.^5^, ^6^, ^7^ This technique provides a very satisfactory view of the surgical field. For the surgeon, the cons are the vibration of the vocal cords and the propulsion of blood and tissue fragments into the operating field. High‐frequency jet ventilation presents serious side effects such as the risk of pulmonary barotrauma, increased in the presence of an obstacle in the upper airways, cutaneous emphysema, and pneumothorax which can lead to the death of the patient.^8^, ^9^

Since 2015, Transnasal Humidified Rapid Insufflation Ventilatory Exchange (THRIVE) has been used in general anesthesia for preoxygenation and difficult exposure airway management. THRIVE use offers new opportunities in laryngology since it was described as a possible exclusive mode of oxygenation during some short surgical procedures. In THRIVE, oxygen is administered through a large nasal cannula at flow rates up to 70 L/min, with a fraction of inspired oxygen (FiO_2_) up to 100%.^10^ THRIVE prolongs the apnoea period while reducing the risk of carbon dioxide (CO_2_) retention and barotrauma.^11^ THRIVE main advantage in laryngeal microsurgery is that it frees up the visual field of the operated site.^12^

To our knowledge, 3 retrospective cohorts (with less than 60 patients each) have demonstrated the safety of THRIVE in laryngeal microsurgery.^13^, ^14^, ^15^ In most studies of THRIVE the use of CO_2_ laser is an exclusion criterion. The risk of ignition contributes at a necessary caution whereas the absence of endotracheal material (cuff, cotton, tube) deprives the procedure of a flammable material in the presence of oxygen (reduced FiO_2_ to 20%–30%) and the laser.^16^

The main objective of our study was to investigate the feasibility of THRIVE in laryngeal microsurgery with cold instruments or CO_2_ laser for different types of laryngeal lesions and pathologies and to identify predictive factors of failure.

## Materials and Methods

For this retrospective study, clinical data of patients who benefited from laryngeal microsurgery under general anesthesia with THRIVE between January 1, 2020 and January 30, 2022 were extracted from patients' medical records. The study received regulatory validation from the Foch Hospital institutional review board (IRB): n°IRB‐00012437. Due to an automated search of patient's nonopposition for medical search using anonymous data from the electronic medical file since 2018, the consent was waived for this analysis.

Inclusion criteria were: all adults who underwent laryngeal microsurgery under general anesthesia with THRIVE as first‐line ventilation method during the procedure. Noninclusion criteria were: panendoscopy for cancers without laryngeal microsurgery and diagnostic biopsies. The noninclusion of panendoscopy was justified by the existing literature supporting its feasibility and safety under THRIVE.^11^

The data collected were as follows: clinical (age, sex, body mass index [BMI], American Society of Anesthesiologist [ASA] score, Mallampati score, smoking assessed in pack‐years [PY]; history [pulmonary and past laryngeal pathologies]; present disease laryngeal history [location in the larynx, type of lesion]; surgical data [procedure, duration of general anesthesia [in minutes], duration of the surgical procedure [in minutes]], recorded minimal peripheral capillary oxygen saturation [SpO_2_], duration of stay and monitoring in the recovery room, use of the CO_2_ laser, occurrence of adverse events and their nature, hospitalization stay in days) and the main outcome that was the abortion of THRIVE and the need for endotracheal intubation during the procedure.

Laryngeal pathologies were divided into 3 subgroups: tumors (squamous cell carcinoma [SCC] and laryngeal papillomatosis); benign lesions (nodules, polyps, cysts, scars, and chronic laryngitis); and laryngeal sequelae (glottic stenosis, laryngeal immobility, vocal fold atrophy).

Surgical procedures were divided into 3 subgroups corresponding to the 3 groups of laryngeal pathologies, respectively: Phonosurgery, cordectomies (type I to VI) (ELS classification), vocal cord medializations, and dilatations of subglottic stenosis. Posterior transverse cordotomies were included in the cordectomy group due to the use of CO_2_ laser to perform a section of the glottic stage. A particular subgroup was made of laryngeal microsurgeries performed using CO_2_ laser.

All patients included in the study received the same type of anesthetic management according to a standardized protocol established by the anesthesia and intensive care teams of our hospital with a continuous measurement of the depth of anesthesia. THRIVE was systematically used as a first‐line oxygenation in all patients undergoing laryngeal surgery.

The Optiflow® system (Fisher & Paykel Healthcare) for THRIVE was used in all patients. At admission in the operative room, the size of the nasal canula was chosen and oxygenation started while the patient was conditioned. Proclive position at 30 to 45° and respiration with closed mouth were recommended before general anesthesia. Propofol (2‐2.5 mg/kg/min at induction for unconsciousness for a total dose of 3‐4 mg/kg) was used in each procedure for induction, combined with remifentanil (2‐2.5 mg/kg) using the Target‐Concentration Infusion (TCI) model.^17^ Rocuronium (0.6 mg/kg and 10 mg re‐injection if longer than 25 minutes) was used for neuromuscular blockade. For some very short procedures, succinylcholine may have been used as curare. The oxygen flow rate administered was 30 L/min for preoxygenation and was increased to 70 L/min when the patient fell into apnea with a FiO_2_ of 100%. When using the CO_2_ laser, FiO_2_ was lowered to 30% and protective measures were installed (wet fields on the face, wet cotton pad (Cotonnoid®; Codman Johnson and Johnson), protective glasses for the operating and anesthetic staffs).

In the event of hypoxia (SpO_2_ < 90%) resistant to immediate measures such as increased oxygen flow, elevated FiO_2_, and airway clearance, or in instances of significant bleeding, the oxygenation strategy was altered. A decision was taken to transition from THRIVE to mechanical ventilation, involving the insertion of an endotracheal tube. This conversion served as the determinant for categorizing outcomes in our study, distinguishing between failure (requiring mechanical ventilation) and success (sustained use of THRIVE throughout the procedure).

Statistical analyses were performed in the R Core Team 2020 software® (R Foundation for Statistical Computing). The significance level, with a possible 2‐sided effect, was set at *α* = 0.05.

## Results

**Figure 1:**
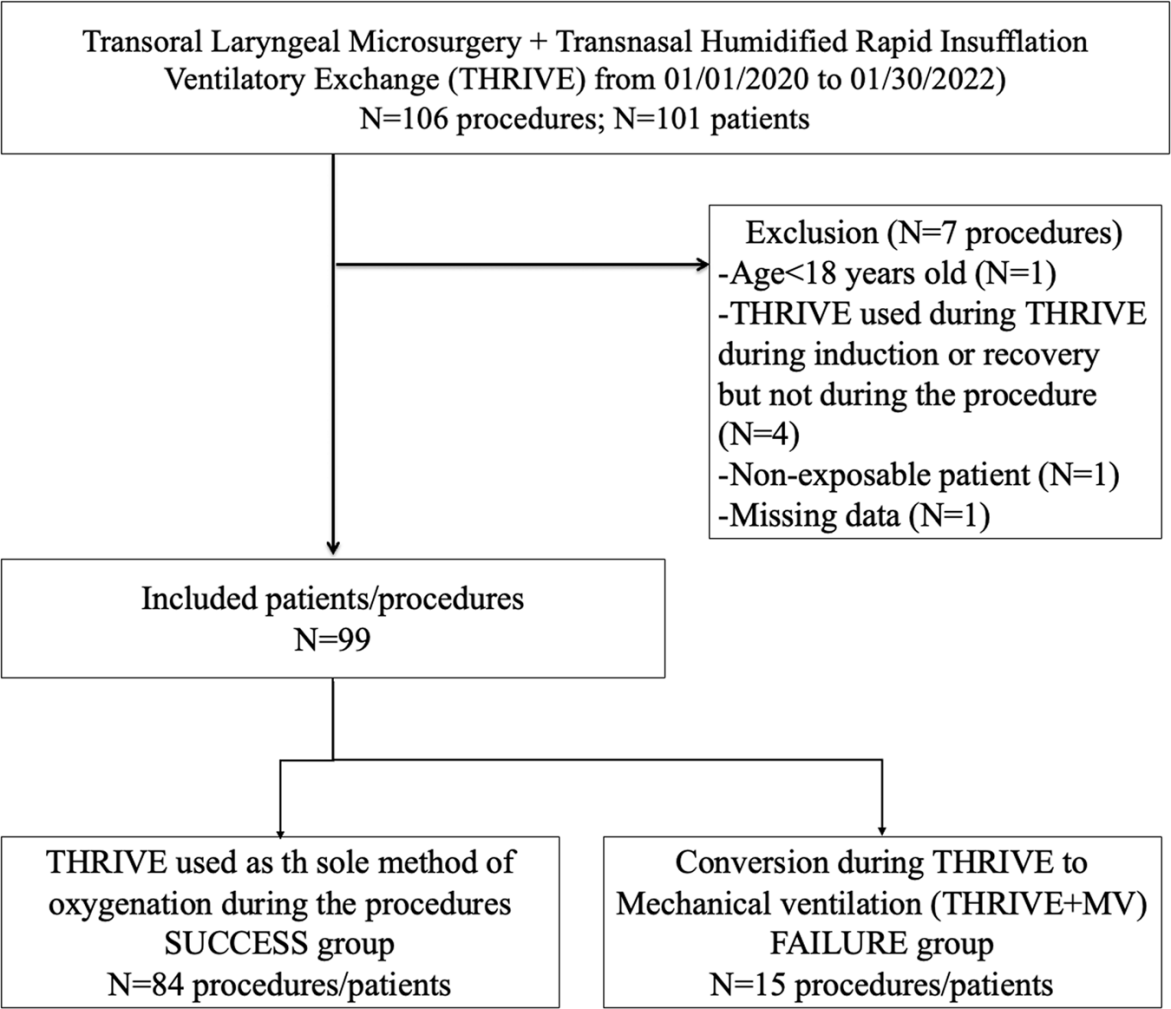
Flow chart. MV, mechanical ventilation; THRIVE, Transnasal Humidified Rapid Insufflation Ventilatory Exchange.

A total of N = 101 patients were included in the study for a total number of 106 procedures performed between January 1, 2020 and January 30, 2022. N = 7 procedures were excluded for the following reasons: age <18 years at the time of surgery (N = 1); use of THRIVE during induction or recovery but not during the procedure (N = 4); nonexposable patient with conversion to external surgery (N = 1); record with missing data (N = 1). Besides, N = 32 panendoscopy procedures for cancer diagnosis were performed under THRIVE in our department. Flow chart is presented in Figure 1.

N = 99 patients who underwent laryngeal microsurgery with THRIVE were analyzed. Patients' general characteristics are presented in Table 1. Pulmonary diseases found in the population were obstructive pulmonary disease (N = 10), pulmonary emphysema (N = 5), past pneumonectomy (N = 1), past pulmonary lobectomy (N = 5), past pulmonary neoplastic lesions (N = 2), Wegener's disease with pulmonary involvement (N = 1), history of pneumothorax (N = 1) and sequelae of severe COVID with hospitalization in intensive care for ventilation (N = 1). No patient had acute or chronic respiratory or cardiac failure. No patient with an ASA 4 score was identified.

**Table 1:** General Characteristics of the Study Population

Characteristics N = 99	Median (N=)/number ±SD (min‐max/percentage)
Age (in years)	57 ± 16.1 (19‐86)
Sex ratio F/M	53/46
BMI (kg/m^2^)	23 ± 3.6 (17‐37)
ASA 1	30 (30%)
ASA 2	49 (49%)
ASA 3	18 (18%)
ASA 4	0 (0%)
Mallampati I	59 (60%)
Mallampati II	34 (34%)
Mallampati III	6 (6%)
Mallampati IV	0
Smoking (pack‐years)	
No	51 (52%)
< 10 PY	3 (3%)
10‐19 PY	7 (7%)
> 20 PY	38 (38%)
Pulmonary pathologies	26 (26%)
Laryngeal pathologies	
Polyps	9 (9%)
Nodules	2 (2%)
Cysts	3 (3%)
Other cord lesions	10 (10%)
Cord atrophy	7 (7%)
Synechiae	1 (1%)
Myxoedema	9 (9%)
Laryngeal immobility	23 (23%)
Subglottic stenosis	2 (2%)
Glottic stenosis	5 (5%)
Malignant tumors	19 (19%)
Laryngeal papillomatosis	9 (9%)
Laryngeal surgery	
Phonosurgery	36 (36%)
Cordectomy and cordotomy	28 (28%)
Medialization‐Dilatation	31(31%)
Use of the CO_2_ laser	19 (19%)

Abbreviations: ASA, American Society of Anesthesiologist; BMI, body mass index; CO_2_, carbon dioxide; PY, Pack‐Years;

THRIVE was used as the sole method of oxygenation in N = 84/99 (84.8%) procedures, N = 15/99 (15.2%) procedures required conversion of the ventilation method to mechanical ventilation with endotracheal intubation.

Hypoxia was the cause of conversion in all the 15 procedures (Table 2). Conversion occurred either before the start of the procedure (N = 5), during the procedure (N = 8) or after the surgical procedure end (N = 2). Failures before the start of the surgical procedure were concomitant with the lowering of the FiO_2_ when using the CO_2_ laser. In 1 case, endotracheal intubation was necessary after the surgical procedure completion because of the persistence of curare action and despite the use of an antagonist, raising up a suspicion for cholinesterase deficiency in this patient.

**Table 2:** Characteristics of Patients Requiring a Change of Oxygenation Method During Surgery

Age	Gender	Type of surgery	SpO_2_ min	Laser use	Causes of THRIVE failure
76	M	Bilateral type II cordectomy	90	Yes	Hypoxia during the procedure
57	M	Medialization	87	No	Hypoxia after the end of the procedure
71	F	Type IV cordectomy	67	Yes	Hypoxia before the start of the procedure
54	H	Type I cordectomy	85	Yes	Hypoxia before the start of the procedure
63	H	Medialization	98	No	Difficulty of exposure
56	H	Type I cordectomy	83	Yes	Hypoxia before the start of the procedure
66	H	Dilatation of subglottic stenosis	84	No	Hypoxia during the procedure
57	H	Resection of papillomatosis lesion	55	No	Hypoxia before the start of the procedure
64	F	Synechia section	88	No	Hypoxia during the procedure
42	H	Medialization	55	No	Hypoxia at induction
67	H	Type II cordectomy	83	Yes	Hypoxia during the procedure
71	F	Type I cordectomy	87	Yes	Hypoxia during the procedure
75	H	Transverse posterior cordotomy	90	Yes	Hypoxia during the procedure
79	H	Type I cordectomy	75	No	Hypoxia after the end of the procedure (delayed action of curar antagonists)
47	H	Transverse posterior cordotomy	94	Yes	Hypoxia before the start of the procedure

Abbreviations: F, female; M, male; SpO_2_ min, minimal peripheral capillary oxygen saturation; THRIVE, Transnasal Humidified Rapid Insufflation Ventilatory Exchange, cordectomy type according to the 2000 classification the European Society of Laryngology.

Characteristics of the success (THRIVE exclusive) group and failure (THRIVE + Mechanical Ventilation (MV)) group are summarized in Table 3.

**Table 3:** Data of the “Exclusive THRIVE” and “THRIVE and Mechanical Ventilation” Groups

Median (N=)/number ±SD (min‐max/percentage)			
Characteristics	THRIVE only n = 84	THRIVE+MV n = 15	Statistics
Age (y.o.)	56 ± 16.7 (19‐86)	64 ± 10.8 (42‐79)	*P* = 0.21
Women	50 (60%)	3 (20%)	*P* = 0.005
Men	34 (40%)	12 (80%)	
BMI (kg/m^2^)	23 ± 3.3 (17‐34)	26 ± 3.7 (22‐37)	*P* = 0.0003
ASA I‐II	71 (84%)	8 (53%)	*P* = 0.006
ASA III‐IV	11 (13%)	7 (46%)	
Pulmonary pathologies	22 (26%)	4 (26%)	*P* = 1.0
Laryngeal pathologies			
Benign lesions	32 (38%)	2 (13%)	*P* = 0.08
Tumor lesions	21 (25%)	7 (46%)	*P* = 0.11
Sequelae	31 (37%)	6 (40%)	*P* = 1.0
Laryngeal surgery			
Phonosurgery	33 (41%)	3 (21%)	*P* = 0.24
Cordectomy/cordotomy	20 (25%)	8 (57%)	*P* = 0.02
Medialization/dilatation	28 (34%)	3 (21%)	*P* = 0.54
Use of the CO_2_ laser	11 (13%)	8 (53%)	*P* = 0.001
Duration of surgery (min)	12.5 ± 6.7 (2‐38)	22.1 ± 12.9 (5‐46)	*P* = 0.002
Total anesthesia time (min)	20.3 ± 8.9 (7‐55)	31.7 ± 15.2 (7‐49)	*P* = 0.003
SpO_2_ min (%)	96 ± 5.0 (70‐100)	87 ± 11.4 (55‐98)	*P* < 0.001
Monitoring time (min) in the ICU	75 ± 34.2 (25‐172)	56 ± 218.4 (36‐906)	*P* = 0.71
Ambulatory hospitalization	68 (80%)	9 (60%)	*P* = 0.09

Abbreviations: BMI, body mass index; CO_2_, carbon dioxide; MV, mechanical ventilation; PICU, Post Interventional Care Unit; THRIVE, Transnasal Humidified Rapid Insufflation Ventilatory Exchange.

Factors that precipitated failures—with their respective odd ratios (OR)—were: an overweight (BMI >25 kg/m^2^) OR = 6.6 [2.9‐35]; ASA score >2 OR = 3.8 [1.7‐18.7]; the use of CO_2_ laser OR = 4.7 [2.3‐24.7]. There was no significant statistical difference between the two groups in terms of monitoring time in the postoperative room the mode of hospitalization (ambulatory or conventional) or the stay duration (*P* > 0.1).

Total anesthesia times were significantly longer in the THRIVE+MV (Failure) group (31.7 ± 15.2 SD vs 20.3 ± 8.9 SD minutes; *P* = 0.003). Surgical times were significantly longer in the THRIVE+MV (Failure) group (22.1 ± 12.9 SD vs 12.5 ± 6.7 SD minutes; *P* = 0.002). SpO_2_ minima were significantly lower in the THRIVE+MV (Failure) group (87% ± 11.4 SD vs 96% ± 5.0 SD; *P* < 0.001). The THRIVE success rate was 88% in patients with lung disease (N = 18/26), 62% in patients with BMI >25 kg/m^2^ (N = 18/29), and 61% in patients with ASA 3 (N = 11/18). Patients with BMI ≥30 kg/m^2^ with successful THRIVE were N = 3 for surgical procedures lasting respectively 10, 15, and 23 minutes.

Data from the CO_2_ laser subgroup are summarized in Table 4. The small sample size did not allow for statistical evidence of predictive criteria for THRIVE failure or adverse events, as none occurred.

**Table 4:** Characteristics of the Laryngeal Microsurgery Subgroup Using the CO_2_ Laser

Median (N=)/number ±SD (Min‐max/percentage)			
	THRIVE only	THRIVE+MV	
Characteristics	N = 11	N = 8	Statistics
Age (median)	65 ± 17.0 (38‐86)	69 ± 10.8 (47‐76)	*P* = 0.59
BMI (median)	23 ± 1.8 (20‐26)	26 ± 4.3 (22‐37)	*P* = 0.004
ASA Score:			
I‐II	9	5	*P* = 0.6
III‐IV	2	3	
Pulmonary pathologies	2	4	*P* = 0.3
Phonosurgery	3	0	
Cordectomy I‐II	7	4	
Cordectomy >II	0	1	
Transverse posterior cordotomy	0	2	
Duration of the surgery (mean)	15 ± 6.8 (9‐32)	20 ± 11.9 (9‐46)	*P* = 0.33
Total duration of general anesthesia (mean)	22 ± 7.1 (14‐36)	32.5 ± 11.9 (19‐49)	*P* = 0.33
SpO_2_ min (mean)	91 ± 5.3 (84‐100)	85 ± 8.2 (67‐94)	*P* = 0.081

Abbreviations: ASA Score, ASA American Society of Anesthesiologist; BMI, body mass index; CO_2_, carbon dioxide; MV, mechanical ventilation; SpO_2_, pulse oxygen saturation; THRIVE, Transnasal Humidified Rapid Insufflation Ventilatory Exchange.

## Discussion

This study demonstrated the feasibility and safety of THRIVE in laryngeal microsurgery, whether performed with cold micro‐instruments or CO_2_ laser. The conditions for performing THRIVE in laryngeal microsurgery were an ASA score ≤2, a BMI ≤25 kg/m^2^ and the absence of use of the CO_2_ laser which imposed a FiO_2_ reduction. These results are coherent with the latest literature review published in 2020 by Huang et al reporting as feasibility criteria for the THRIVE technique in laryngeal microsurgery: short‐duration surgeries (<30 minutes), patients with an ASA score ≤2 and a BMI ≤25 kg/m^2^ ^18^ and with a recent study published in 2023 with a series of 172 Transoral Laser Microsurgeries (TLM) with THRIVE.^19^ To date, the lack of data concerning populations with an ASA score >2, a BMI >25 kg/m^2^ or associated pulmonary pathologies could not allow us to confirm the feasibility of the THRIVE technique for them. This study performed in filling the blank and allowed to enrich the current literature regarding these patients with a THRIVE success rate of 88% in patients with a present a pulmonary pathology (N = 18), of 62% in patients with a BMI >25 kg/m^2^ (N = 29) and 61% in patients with an ASA 3 score (N = 18).

Due to the limited number of patients, we were unable to identify other risk factors for failure. In addition to this limitation, a selection biases must be acknowledged: some cases of early failure (during the preoxygenation period) of THRIVE might not have been identified and might not appeared in this cohort. Similarly, THRIVE might not have been applied as systematically as we wanted to patients with very high BMI or respiratory failure. This study did not provide information for the use of THRIVE in ASA 4 patients as none were included. These limitations are to be considered as deviations from the protocol. Focusing on situations in which patients were considered eligible for THRIVE and actually benefited from this technique during anesthesia, our data represent the conditions of real clinical practice in an expert center in Laryngology.

Another limitation of the study was the lack of capnia monitoring during anesthesia. THRIVE is an apnea oxygenation technique that granted a limited increase in CO_2_ blood pressure as reported by Gustafsson et al in patients with a BMI <30 kg/m^2^ and without major organ failure.^11^ However, capnia monitoring is likely to improve the management and control of ventilation in providing a thinner evaluation of the respiratory system during procedure. Furthermore, the determination of apnea relied on visual examination, introducing a potential for variability in its monitoring. This is why the duration of anesthesia and surgery was favored as more objective criteria. The elevation in capnia is an undesirable outcome associated with this technique, a consideration non particularly detrimental for brief procedures such as cordectomies. Notably, none of the patients exhibited any adverse effects attributable to hypercapnia. However, adherence to good clinical practice should necessitate the monitoring of capnia as part of a comprehensive evaluation.

Although BMI was significantly higher in the THRIVE failure group compared to the exclusive THRIVE success group, THRIVE seemed feasible, especially in obese patients. In 2018, Lee et al reported the success of THRIVE in a morbidly obese patient for a short procedure (14 minutes long)^20^ as we reported it in N = 3 patients.

No side effect was reported with THRIVE. In the event of THRIVE failure, monitoring time in recovery room was not affected. This should be considered in the light of the serious side effects linked to barotrauma (pneumothorax, pneumomediastinum, death) reported with high‐frequency jet ventilation.^21^ Remarkably, capnia was not monitored in this study. On the one hand, during THRIVE, capnia is not systematically monitored, and, on the other hand, apneic ventilation techniques are more prone to hypercapnia. This must be taken into account in the duration of postoperative monitoring, although a recent study of 40 patients suggests that THRIVE can shorten the duration of postoperative monitoring.^22^

In laryngeal cancers, data reported the use of THRIVE are scarce. Yet, freeing the surgical site from obstacle appears crucial for the gesture accuracy and therefore for the disease control in a minimal invasive approach.^14^, ^23^

TLM became the standard surgical approach for the treatment of early glottic SCC, the alternative is radiation therapy.^24^ The use of the laser in laryngeal oncology to treat T1, T2, and some selected T3 of the glottic stage grants results equivalent to radiotherapy and satisfactory vocal results.^5^, ^25^, ^26^ Transoral cordectomies superior to type II, according to the classification the European Society of Laryngology published in 2000,^27^ with a greater risk of bleeding, are particularly suitable for the use of the laser to allow haemostasias to be achieved at the same time as the resection. No study investigated the impact of glottic plane obstruction by a tube on the accuracy of the procedure and the control of cancerous disease. The vision of the operating field may be reduced by about 50%. The vision of the posterior or even middle part of the vocal folds is compromised by the endotracheal tube. This difficulty disappears under THRIVE, which is its main interest. The laser is also used in the management of laryngeal sequelae (laryngeal paralysis and glottis stenosis). These procedures are performed on the posterior glottis such as transverse posterior cordotomy and median arytenoidectomy.^28^, ^29^

No ignition occurred in our series. In fact, THRIVE offers additional safety features: the absence of flammable material in the larynx and trachea. In these new conditions permitted by the THRIVE, we could consider modifying the safety parameters, in particular keeping a higher FiO_2_ in order to lengthen the duration of the apnea. One study tested these parameters on porcine larynxes. No fire occurred, even at 100% FiO_2_ and 5 W Laser in the absence of cotton and tube.^16^ Nevertheless, protective measures must be always applied when using laser: first and foremost, the reduction of the FiO_2_ to 30% immediately before and during the laser procedure.^30^ All safety measures used during TLM must be applied regardless of the ventilation mode. In our series, no incident occurred using the usual safety measures when using the Laser. The small number of patients studied did not allow us to identify a predictive factor of failure other than the lowering of FiO_2_. ASA score 3 or 4, the long duration of preoxygenation before the apnea period and the speed of FiO_2_ reduction appeared normally as possible predictive risk factor of failure, although not significant. This corrective measure was the main reason identified for THRIVE failure. In the current state of science, the possibility of using a higher FiO_2_ does not seem feasible.^16^, ^31^

If the patients tolerate the lowering of the FiO_2_, a mixed procedure can be considered. In the case of a posterior glottic lesion, the first part of the procedure could be performed with THRIVE, then as soon as the exposure of the surgical field no longer justifies it, the rest of the procedure could be performed with the placement of an endotracheal tube.

## Conclusion

In conclusion, this study confirmed the feasibility of endoscopic laryngeal microsurgery using THRIVE, including when the CO_2_ laser is used. Age, obesity, ASA score, and history of pulmonary pathology were not found to be predictive of failure. However, in obese patients and those with a high ASA score, it should preferably be used for short duration procedures. This mode of oxygenation is applicable to different types of surgical procedures, whether it be voice surgery, vocal cord medialization or TLM. It allows the surgeon a better access to the posterior glottis, a unique visual control and access to the entire surgical field. The use of the CO_2_ laser is feasible under suitable conditions; however, the probability of apnea non‐tolerance is increased due to the lowering of the FiO_2_.

## Author Contributions

**Tiffany Rigal**, **Stéphane Hans**, **Robin Baudouin**, **Morgan Le Guen**, study concept and design; **Tiffany Rigal**, **Robin Baudouin**, **Marta Circiu**, **Lise Crevier‐Buchman**, **Florent Couineau**, acquisition, analysis, or interpretation of data; **Jérôme Lechien**, **Marta Circiu**, **Florent Couineau**, drafting of the manuscript; **Tiffany Rigal**, **Robin Baudouin**, **Morgan Le Guen**, **Stéphane Hans**, critical revision of the manuscript for important intellectual content.

## Disclosures

### Competing interests

None.

### Funding source

None.
